# Exploratory Small RNA Sequencing in Localized Versus Locally Advanced Prostate Cancer: Putative Roles of CLTC and CDK6 as Downstream Targets of Differentially Expressed MicroRNAs

**DOI:** 10.3390/ph19081141

**Published:** 2026-07-23

**Authors:** Jae Heon Kim, Ahrim Moon, Miho Song, Kwang Woo Lee, Soo Min Suh, Hui Ji Kim, Luis Alfonso Pefianco, Kevin Andrean, Kisoo Lee, Seongho Ryu, Yun-Seob Song

**Affiliations:** 1 Department of Urology, Soonchunhyang University School of Medicine, Seoul 04404, Republic of Korea; piacekjh@hanmail.net (J.H.K.); miho@schmc.ac.kr (M.S.); 2 Department of Pathology, Soonchunhyang University School of Medicine, Bucheon 14584, Republic of Korea; armoon@schmc.ac.kr; 3 Department of Urology, Soonchunhyang University School of Medicine, Bucheon 14584, Republic of Korea; urolkw@schmc.ac.kr; 4 Soonchunhyang Institute of Medi-Bio Science (SIMS), Soonchunhyang University, Cheonan 31151, Republic of Korea; niceolvia@naver.com (S.M.S.); khjee1932@gene2us.com (H.J.K.); kevinandrean@sch.ac.kr (K.A.); 5 Department of Integrated Biomedical Science, Soonchunhyang University, Cheonan 31151, Republic of Korea; 6 Department of Urology, Dong-A University College of Medicine, Busan 49201, Republic of Korea

**Keywords:** prostate cancer, microRNA, locally advanced prostate cancer, CLTC, CDK6, exploratory pilot study

## Abstract

**Background/Objectives:** Prostate cancer (PCa) remains a leading cause of cancer-related mortality in men. While localized PCa carries a favorable prognosis, progression to locally advanced disease substantially worsens outcomes. There is an unmet need for molecular markers that reliably distinguish indolent from aggressive tumors. MicroRNAs (miRNAs), small non-coding RNAs that post-transcriptionally regulate gene expression, are implicated in cancer development and progression, and their expression profiles may serve as putative biomarkers of disease stage. **Methods:** We performed small RNA sequencing on formalin-fixed paraffin-embedded (FFPE) prostate tumor tissues from nine patients (five localized, four locally advanced-stage). Differentially expressed miRNAs were identified using a bioinformatics pipeline (fold-change ≥ 2, *p* < 0.05). Target gene prediction, KEGG pathway enrichment, and miRNA–mRNA interaction network analyses were conducted, followed by in silico validation using two independent public mRNA datasets (GSE46602 and GSE21034, a subseries of GSE21032). **Results:** Seven unique mature miRNAs were differentially expressed between localized and locally advanced PCa. Five miRNAs (miR-145-5p, miR-205-5p, miR-206, miR-24-1-5p, and hsa-miR-3648) were downregulated in locally advanced tumors, while hsa-miR-625-3p and miR-324-5p were upregulated. Network analysis identified CLTC and CDK6 as putative downstream targets co-targeted by multiple downregulated miRNAs. Independent validation using GSE46602 and GSE21034 consistently demonstrated significantly higher expression of CLTC and CDK6 in locally advanced prostate cancer following Benjamini–Hochberg correction. **Conclusions:** This exploratory pilot analysis identified several miRNAs that differ between localized (pT2) and locally advanced (pT3–T4/N+) prostate cancer. Independent validation across two publicly available mRNA cohorts supports CLTC and CDK6 as candidate stage-associated genes. However, these findings remain hypothesis-generating and require validation in larger prospective cohorts together with functional confirmation before biomarker or therapeutic claims can be made.

## 1. Introduction

Prostate cancer (PCa) is the second most commonly diagnosed malignancy in men worldwide, with approximately 1.4 million new cases and over 375,000 deaths estimated in 2020 [1]. Localized disease is generally manageable with surgery or radiotherapy; however, progression to locally advanced or metastatic stages markedly worsens survival, and treatment at this point becomes palliative [2,3]. A central challenge in uro-oncology is accurately predicting which tumors will remain indolent and which will progress.

Conventional risk-stratification tools—serum prostate-specific antigen (PSA), Gleason score, and pathological stage—have well-recognized limitations, particularly for patients in the intermediate-risk category (PSA 4–10 ng/mL) [4]. Accordingly, there is considerable interest in molecular biomarkers capable of improving prognostic accuracy and guiding individualized treatment.

MicroRNAs (miRNAs) are ~22-nucleotide endogenous non-coding RNAs that regulate gene expression by binding target mRNAs, inducing their degradation or translational repression. Because each miRNA can modulate multiple target genes, miRNAs collectively influence a broad spectrum of cancer-related processes including proliferation, apoptosis, angiogenesis, and metastasis [5]. Their stability in clinical specimens—including tissue, blood, and urine—makes them attractive minimally invasive biomarker candidates.

Aberrant miRNA expression is a hallmark of PCa and reflects the oncogenic state. Tumor-suppressive miRNAs are frequently downregulated in PCa, relieving repression of oncogenic target genes, while oncogenic miRNAs (oncomiRs) are upregulated and suppress tumor suppressor pathways. For example, members of the miR-17~92 cluster (miR-17, miR-20a/b, miR-106a) are associated with advanced stage and earlier biochemical recurrence [6,7,8,9,10], whereas miR-153 upregulation correlates with high Gleason score and poor survival [10]. High-throughput sequencing studies have further demonstrated broad miRNA downregulation during PCa progression, with miR-1, miR-143, miR-145, miR-205-5p, and miR-221/222 consistently reduced in advanced tissues. Conversely, hsa-miR-6715b-3p has been found markedly overexpressed across increasing PCa stages, potentially via PTEN pathway suppression.

Despite this growing body of evidence, relatively few studies have directly compared miRNA profiles between localized and locally advanced PCa tumors. PCa tumors. Such comparisons are essential to identify stage-specific molecular changes that may serve as both biomarkers and therapeutic targets. In this study, we profiled miRNA expression in FFPE prostate tumor tissues from patients with localized versus advanced PCa using next-generation sequencing, identified differentially expressed miRNAs (DE-miRNAs), and explored their putative target genes, enriched pathways, and interaction networks. In silico analysis using public datasets was performed to validate key findings.

## 2. Results

### 2.1. Patient Profiles

Clinical and pathological characteristics are summarized in Table 1. The localized (*n* = 5) and locally advanced (*n* = 4) groups were comparable in age (mean 65.0 vs. 73.0 years, *p* > 0.05) and preoperative PSA (22.8 vs. 21.2 ng/mL, *p* > 0.05). As expected, pathological stage (pT2 vs. pT3–T4; *p* < 0.01) and Gleason grade (mean 2.8 vs. 4.3; *p* = 0.03) differed significantly between groups. Two of the four patients with locally advanced prostate cancer experienced biochemical recurrence during follow-up. Of these, one subsequently developed clinical progression and ultimately died of prostate cancer.

### 2.2. Differentially Expressed miRNAs

**Figure 1:**
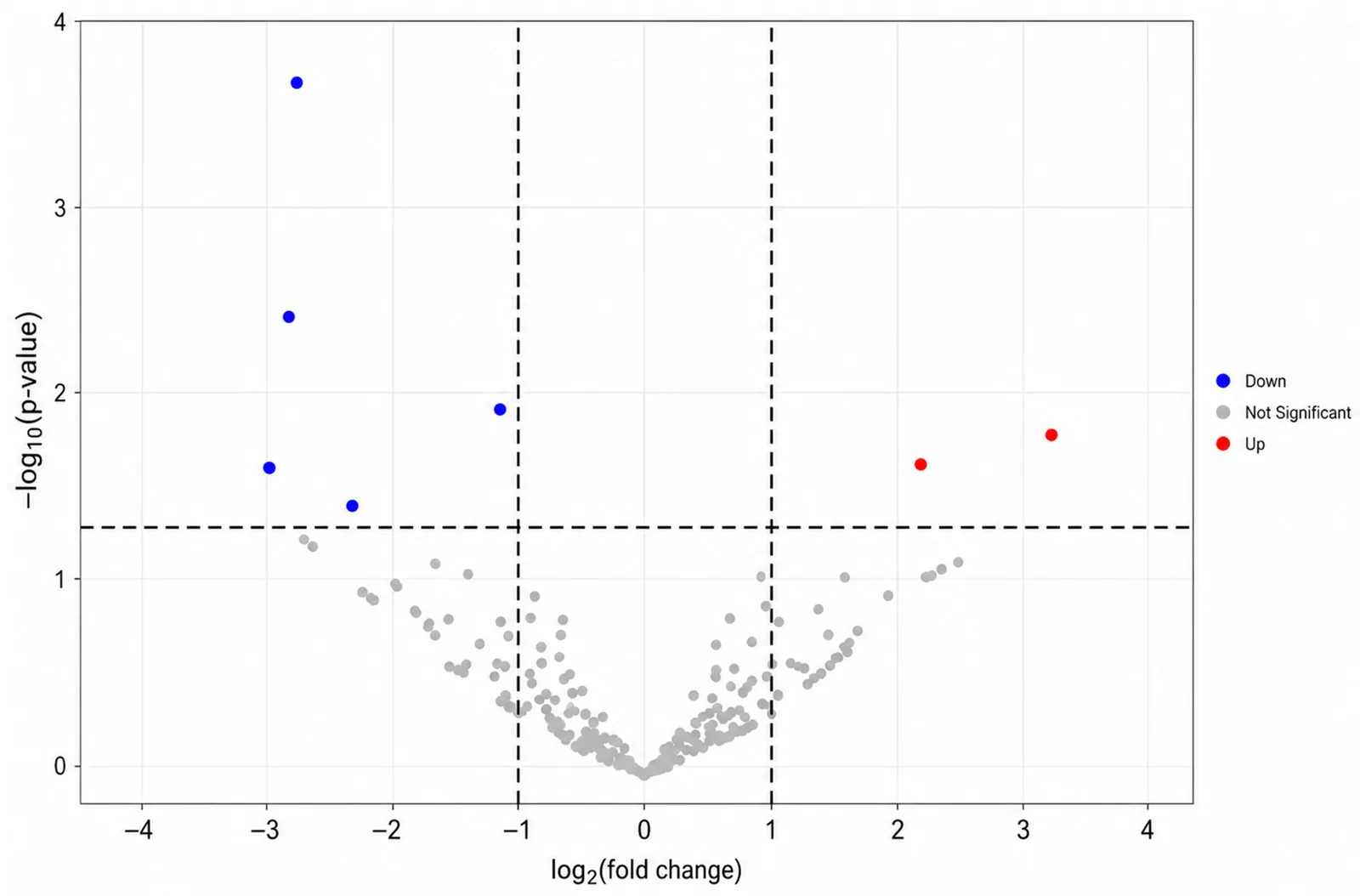
Differential miRNA expression between localized and advanced prostate cancer. Volcano plots and hierarchical clustering of differentially expressed miRNAs according to prostate cancer stage (fold change ≥ 2 and *p* < 0.05).

**Figure 2:**
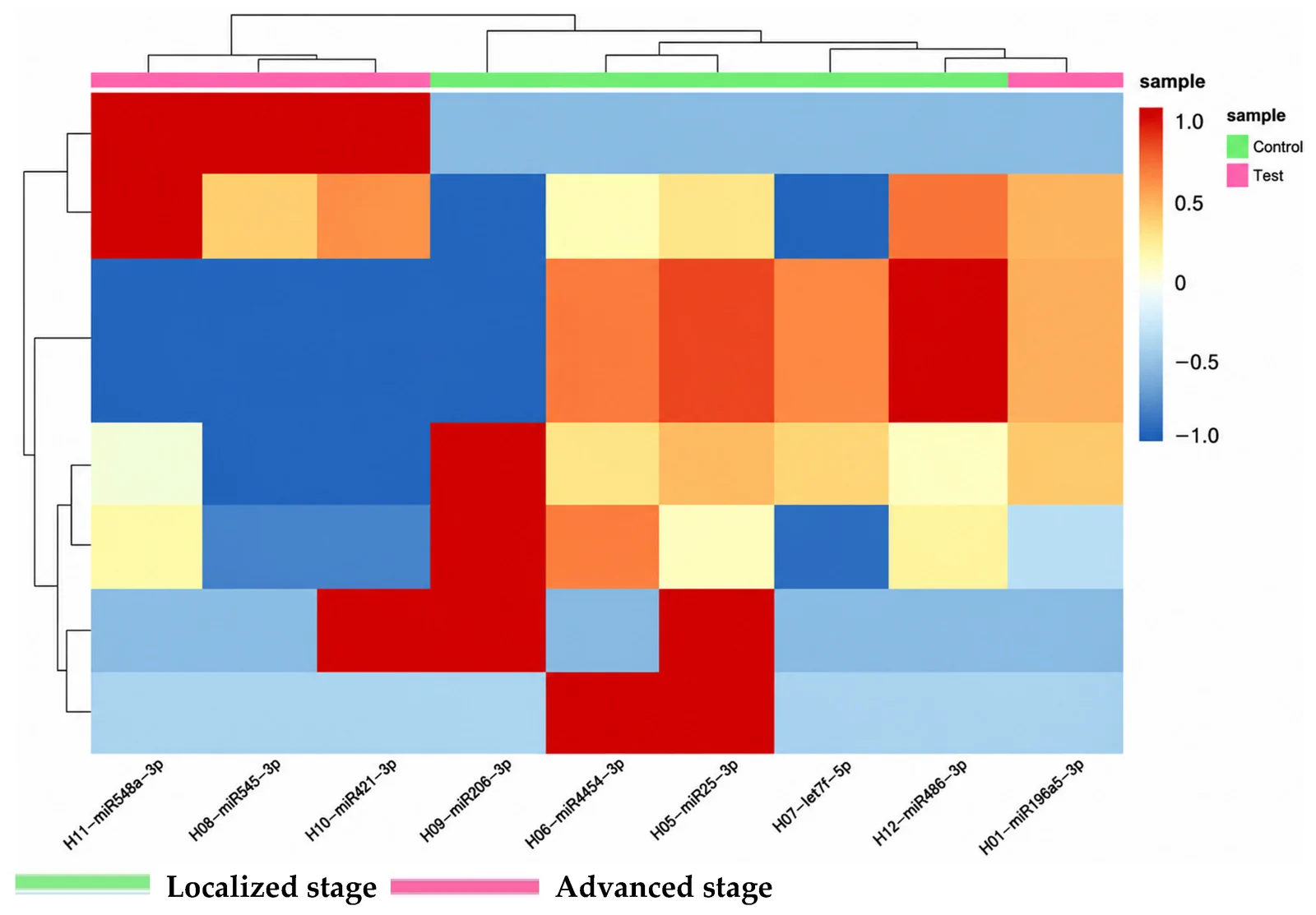
Differentially expressed miRNAs in prostate cancer tissues and heatmap. Unsupervised hierarchical clustering of samples based on the 7 most significant differentially expressed mature miRNAs. The heatmap shows Z-scores to preserve absolute expression level information. This figure is a visualization of the discovery dataset only and does not constitute independent validation of a diagnostic signature. One locally advanced sample (ISUP GG3, pT3a) clusters with the localized group, likely reflecting intermediate-grade biology.

Sequencing identified seven unique mature DE-miRNAs between localized and locally advanced PCa (*p* < 0.05; Table 2; Figure 1). False discovery rate (FDR)-adjusted *p*-values were additionally calculated using the Benjamini–Hochberg procedure to account for multiple testing. The seven mature miRNAs remained significant after adjustment and were therefore retained for downstream analyses. Five unique mature miRNAs—miR-145-5p, miR-205-5p, miR-206, miR-24-1-5p, and hsa-miR-3648 (detected from two precursor loci but representing one mature sequence)—were downregulated in locally advanced tumors, whereas hsa-miR-625-3p and miR-324-5p were upregulated. Unsupervised hierarchical clustering showed distinct grouping in the discovery dataset (Figure 2); one locally advanced sample (ISUP GG3, pT3a) clustered with the localized group. The greatest fold changes were for miR-205-5p and miR-145-5p (both > 4-fold lower) and hsa-miR-625-3p (~3-fold higher). These findings are broadly consistent with previous reports of stage-associated miRNA dysregulation in prostate cancer.

### 2.3. Pathway and Disease Ontology Analysis

**Figure 3:**
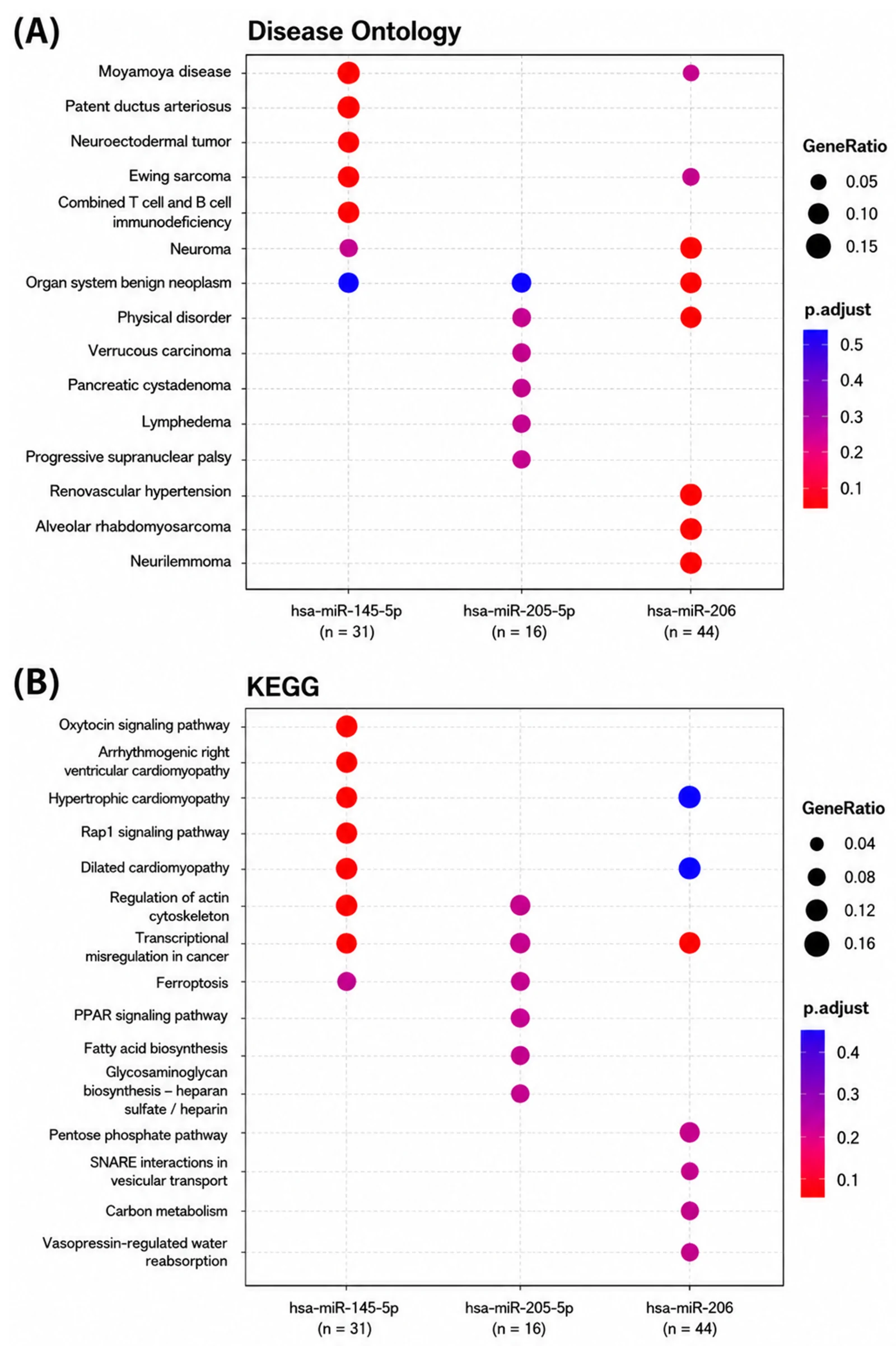
MicroRNA pathway and disease ontology analyses according to pathological stage of prostate cancer tissues: (**A**): Disease ontology analysis. Disease ontology terms are presented for completeness and should be interpreted cautiously because several enriched categories have limited direct biological relevance to prostate cancer. Detailed results are provided in Supplementary Table S3. (**B**): KEGG pathway. The oxytocin signaling pathway was enriched among predicted target genes associated with miR-145-5p. Regulation of actin cytoskeleton was enriched among predicted target genes associated with miR-205-5p. Transcriptional misregulation in cancer was enriched among predicted target genes associated with miR-206.

To explore the biological impact of the DE-miRNAs, we performed target gene prediction and pathway enrichment analysis for both the downregulated and upregulated miRNAs separately. For the six downregulated miRNAs, KEGG analysis identified enrichment in PI3K-Akt, MAPK, focal adhesion, and actin cytoskeleton regulation pathways, consistent with tumor-suppressive functions. For the upregulated miRNAs (hsa-miR-625-3p and miR-324-5p), predicted target genes were enriched in cell cycle arrest and apoptosis pathways, consistent with oncomiR activity. Disease Ontology terms with weak prostate cancer relevance (Moyamoya disease, Ewing sarcoma, pancreatic cystadenoma) are provided in Supplementary Table S3 only. These terms should be interpreted cautiously because they arise from shared gene-set enrichment rather than disease-specific biological associations with prostate cancer. Figure 3 uses log_10_-scale color-coding for *p*-values. Full enrichment tables with gene counts, background counts, raw and adjusted *p*-values, and target gene lists are in Supplementary Table S3.

### 2.4. miRNA–Target Gene Interaction Network

**Figure 4:**
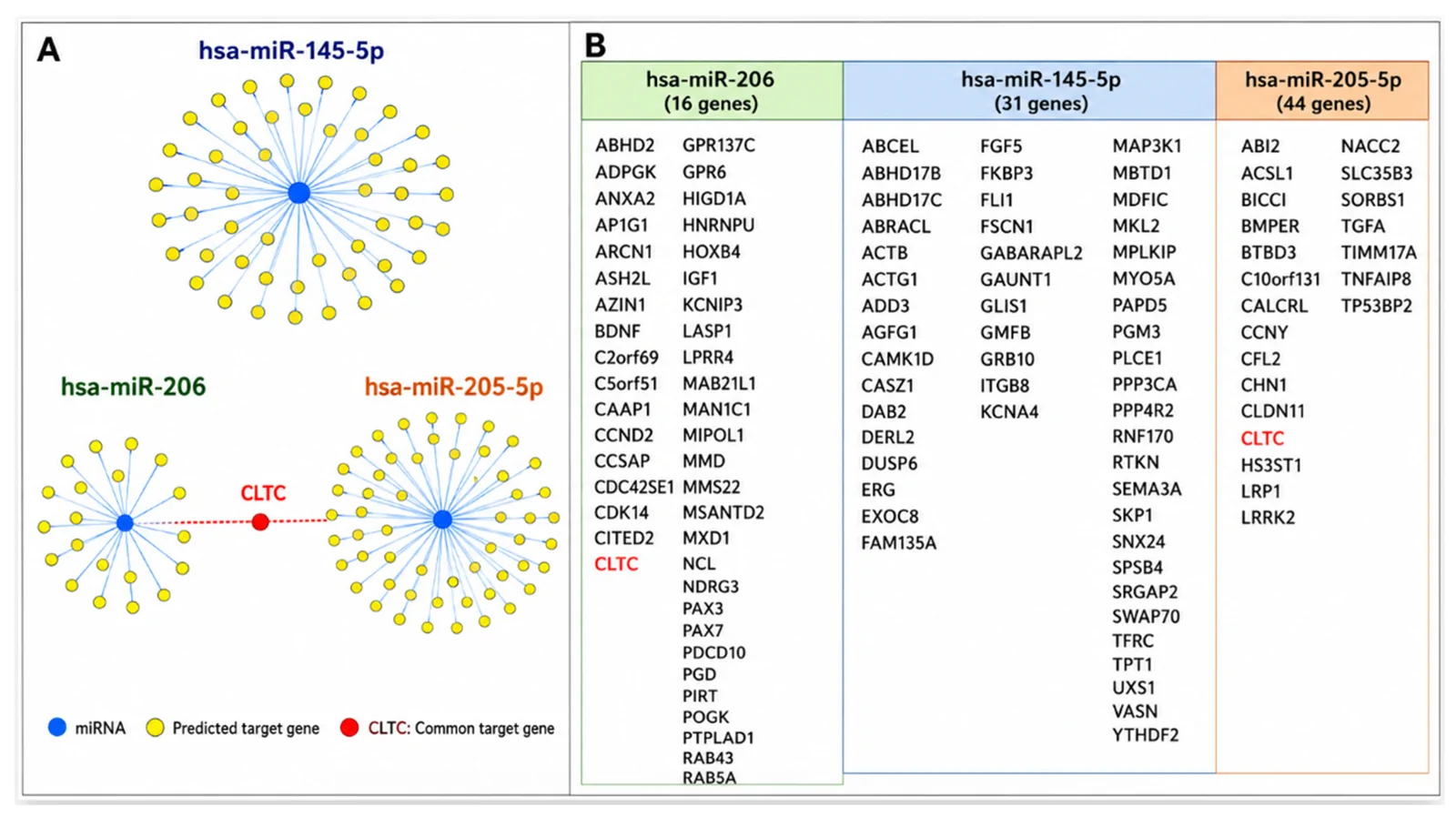
Predicted microRNA–target gene interaction networks based on differentially expressed microRNAs identified between localized and locally advanced prostate cancer tissues: (**A**) Network representation of predicted target genes for hsa-miR-206, hsa-miR-145-5p, and hsa-miR-205-5p. MicroRNA hubs are depicted as blue nodes, whereas predicted target genes are depicted as yellow nodes. hsa-miR-206, hsa-miR-145-5p, and hsa-miR-205-5p were predicted to interact with 83, 51, and 22 target genes, respectively. (**B**) List of predicted target genes for each microRNA. Clathrin heavy chain (CLTC) was identified as a shared target gene co-targeted by hsa-miR-206 and hsa-miR-205-5p and is highlighted in red. Detailed target-gene lists, interaction evidence classifications, and network metrics are provided in Supplementary Table S2 for improved readability.

An interaction network of the six downregulated miRNAs and their validated or high-confidence predicted targets revealed several highly connected target genes (convergence nodes) co-targeted by multiple miRNAs (Figure 4). The network was extended to include all six downregulated miRNAs; the evidence class for each interaction (miRTarBase experimental vs. TargetScan computational) is indicated in Figure 4 and Supplementary Table S2. CLTC (Clathrin Heavy Chain) was identified as a convergence node sharing predicted binding sites for both miR-205-5p and miR-206. CDK6 was predicted as a target of miR-24-1-5p and miR-145-5p. Network degree centrality values for all target genes are provided in Supplementary Table S2. The convergence of multiple downregulated miRNAs on these putative targets suggests that their collective loss may de-repress these genes in locally advanced PCa; experimental validation is required to confirm these relationships. For readability, detailed target-gene lists, interaction evidence classes, and network centrality metrics are provided in Supplementary Table S2.

### 2.5. In Silico Validation of Hub Genes

**Figure 5:**
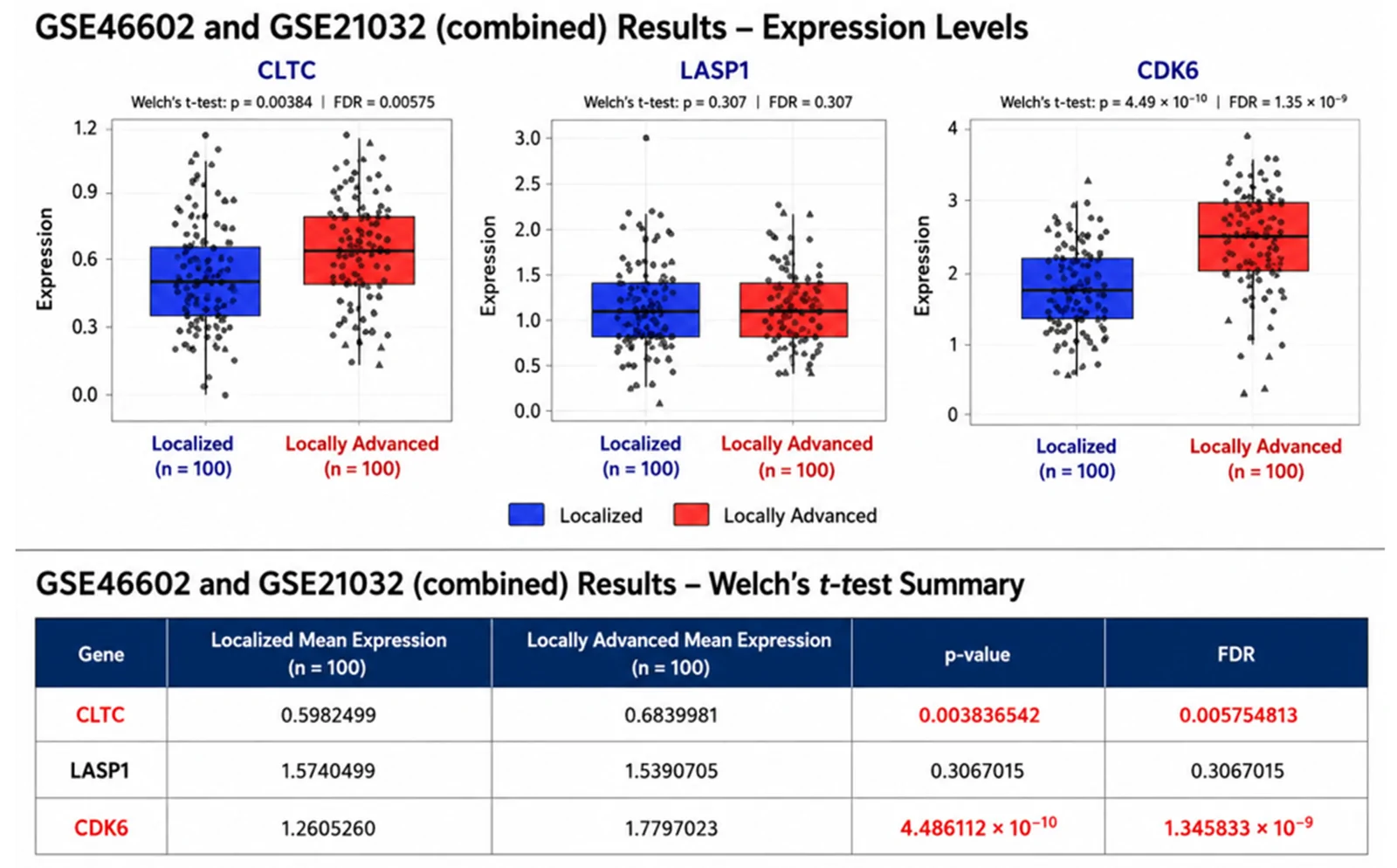
The investigation of differentially expressed microRNAs associated with prostate cancer progression as potential biomarkers and therapeutic targets. Validation analyses were performed separately in two independent datasets, GSE46602 and GSE21034 (a subseries of GSE21032). They showed that CLTC and CDK6 expression was significantly higher in locally advanced prostate cancer compared to localized cancer in GEO datasets (FDR < 0.01). LASP1 did not differ significantly between groups in either dataset.

To validate the clinical relevance of the identified hub genes, we analyzed expression data from GSE46602 and GSE21034 (a subseries of GSE21032) (Figure 5). GSE46602 (Affymetrix HG-U133 Plus 2.0) and GSE21034 (Affymetrix Human Exon 1.0 ST) were generated on different microarray platforms and were therefore quantile-normalized and analyzed separately rather than merged. CLTC and CDK6 were significantly overexpressed in locally advanced compared with localized prostate cancer in both datasets after Benjamini–Hochberg correction (all adjusted *p* < 0.05). LASP1 did not differ significantly between groups in either dataset (adjusted *p* > 0.05). The concordant direction of effect across two independently analyzed cohorts profiled on different platforms supports CLTC and CDK6 as candidate stage-associated genes.

## 3. Discussion

This exploratory pilot study identified candidate miRNAs that differ in expression between localized (pT2) and locally advanced (pT3–T4/N+) prostate cancer in a cross-sectional comparison of nine archival FFPE specimens. Because the design compares two pathological stage groups rather than following individual tumors over time, findings reflect stage-associated differences and should not be interpreted as evidence of dynamic tumor progression. These findings are consistent with accumulating evidence that loss of miRNA-mediated tumor suppression is associated with PCa stage advancement [6,7], but require independent validation before conclusions can be drawn.

**miR-206** was ~2.7-fold lower in advanced tumors. Wang et al. demonstrated that miR-206 suppresses PCa cell proliferation and invasion by targeting CXCL11, thereby attenuating pro-tumorigenic chemokine signaling [11]. Its downregulation was also identified in large-scale systems analyses as a stage-associated miRNA [6], reinforcing its potential as a candidate tissue marker.

**miR-205-5p** was ~3.8-fold reduced in advanced tumors. MiR-205 maintains epithelial identity by suppressing ZEB1/ZEB2 and inhibiting epithelial-to-mesenchymal transition (EMT). Yamada et al. demonstrated that miR-205-5p directly targets HMGB3, an oncogene linked to PCa invasion and poor prognosis [12]. Loss of miR-205 in advanced tumors likely unleashes EMT and pro-metastatic programs. The detectability of miR-205 in urinary exosomes suggests potential future utility as a non-invasive marker pending prospective validation [13,14,15].

**miR-145-5p** showed the greatest downregulation (~4-fold). As a broad-spectrum tumor suppressor, miR-145 targets WIP1 (PPM1D), c-Myc, SOX2, and ADAM17, among others. Clinical studies confirm that low miR-145 expression associates with advanced stage, biochemical recurrence, and reduced overall survival in PCa [16,17,18]. miR-145 mimics have demonstrated preclinical efficacy in inducing apoptosis and sensitizing PCa cells to therapy; translational potential requires further investigation.

**miR-24-1-5p** was ~2.4-fold lower in advanced tumors. Lynch et al. reported that miR-24 suppresses PCa cell cycle progression by downregulating CDC27 and CDKN1B/p27 [19]; its loss may therefore contribute to unchecked proliferation in locally advanced disease.

**miR-3648-1 and miR-3648-2** are derived from small Cajal body-specific RNA (scaRNA) fragments rather than canonical miRNA genes. Their biological function in cancer is poorly characterized; they are reported here for completeness.

Regarding the upregulated miRNAs, miR-625-3p was approximately three-fold elevated in locally advanced prostate cancer. The biological role of miR-625-3p in prostate cancer remains incompletely characterized, and therefore the present findings should be considered exploratory. Additional experimental and clinical studies are required to clarify its functional significance and potential role in disease progression. **miR-324-5p** was ~2-fold elevated. It has been reported as a serum diagnostic biomarker for PCa and as a promoter of tumor cell invasion in gastric cancer [20], pointing to a potential pro-tumorigenic role in advanced PCa as well.

A key finding of this study is the identification of **CLTC** and **CDK6** as hub genes in the miRNA target network, with in silico validation confirming their overexpression in advanced PCa. CLTC encodes the structural coat protein of clathrin-coated vesicles and mediates receptor-mediated endocytosis. Its upregulation—consequent to the loss of miR-205 and miR-206—could enhance recycling of growth factor receptors (e.g., EGFR, TGF-β receptors), sustaining downstream PI3K/AKT and MAPK signaling and promoting tumor cell proliferation and invasion. CDK6 promotes G1-to-S phase transition by phosphorylating the retinoblastoma protein (RB) and has been associated with higher Gleason grade, advanced pathological stage, and resistance to androgen deprivation therapy [21,22]. CDK4/6 inhibitors are currently under investigation in combination with androgen-targeting agents for advanced PCa, further underscoring the clinical relevance of CDK6 as a therapeutic target.

These preliminary findings suggest directions for future research. Circulating forms of several miRNAs (miR-205, miR-145) have been detected in blood and urine; their utility as non-invasive markers for locally advanced PCa requires prospective validation with matched liquid biopsy samples and is not supported by the current dataset. Should functional studies confirm the tumor-suppressive roles of these miRNAs, replacement therapy using synthetic miRNA mimics may warrant future evaluation; no delivery strategies or clinical claims are made here. Comparisons with existing genomic classifiers (e.g., Decipher, Prolaris) require an adequately powered validation cohort and are beyond the scope of this pilot study.

**Limitations** include the small sample size (*n* = 9), which limits generalizability, statistical power, and the ability to adjust for confounders such as age and Gleason grade. Age and Gleason score differed between groups (mean 65 vs. 73 years; ISUP GG1–2 vs. GG3–5); miRNA differences may partly reflect grade rather than stage. The locally advanced group comprised pT3–T4 tumors without distant metastases; future studies should include metastatic specimens. Tumor purity and stromal contamination are potential confounders: miR-145, miR-205, and miR-206 are expressed in stromal, smooth-muscle, and epithelial cells; estimated tumor cellularity per sample is provided in Supplementary Table S1; laser-capture microdissection is recommended for future work. The long archival period (2002–2012) may introduce RNA degradation effects; DV200 metrics are provided. qRT-PCR validation of selected miRNAs in the same and an independent cohort is a critical next step. No biomarker performance metrics (ROC, AUC) are provided, as *n* = 9 does not support such analyses. Future studies should include larger, adequately powered validation cohorts and multivariable analyses to distinguish the independent contributions of pathological stage and tumor grade to the observed miRNA expression patterns. These findings also support the growing importance of improving molecular risk stratification in prostate cancer. Although clinicopathological parameters remain the cornerstone of treatment decision-making, additional molecular biomarkers may further refine individualized risk assessment and complement current clinical management strategies [23].

## 4. Materials and Methods

### 4.1. Patient Recruitment and Sample Collection

**Figure 6:**
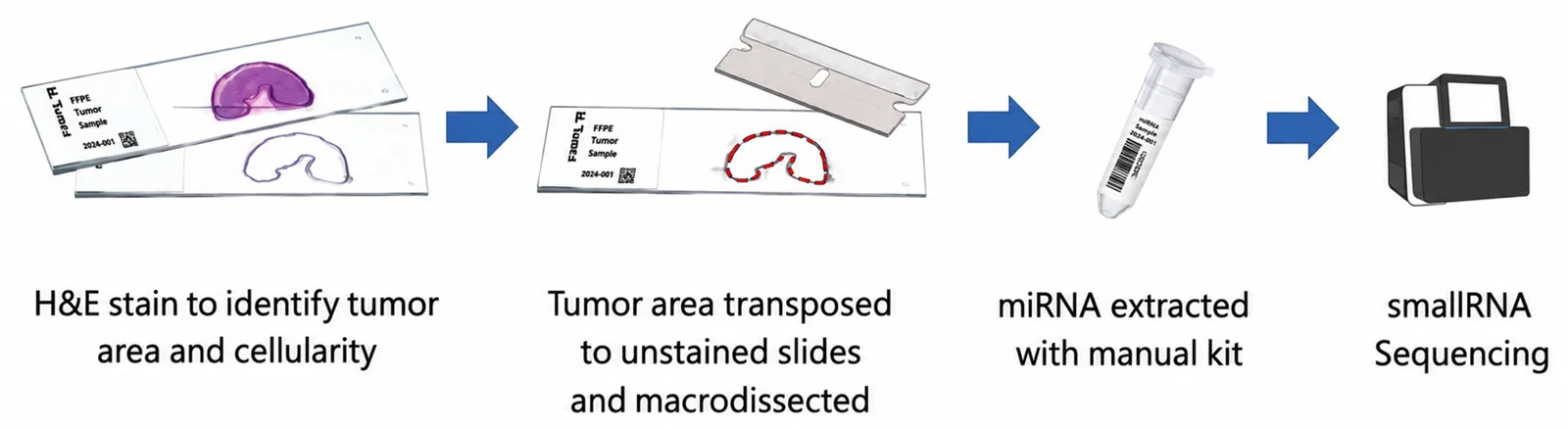
Schematic view of sample collection.

This study included nine male patients with histologically confirmed prostate adenocarcinoma who underwent radical prostatectomy at Soonchunhyang University Hospitals between 2002 and 2012. Five patients had localized PCa (organ-confined, pT2) and four had locally advanced PCa (pT3–T4 and/or lymph-node-positive disease without distant metastasis). An individual patient-level table with exact pT stage, pN status, Gleason score, ISUP Grade Group, margin status, extracapsular extension, seminal vesicle invasion, tumor cellularity (%), FFPE block year, DV200, follow-up duration, biochemical recurrence, and cancer-specific mortality is provided in Supplementary Table S1. FFPE tumor specimens were archived in pathology departments; representative tumor regions were identified on hematoxylin and eosin (H&E)-stained sections by board-certified pathologists (Figure 6). Clinicopathological data were retrieved from medical records. The study was approved by the Institutional Review Boards of Soonchunhyang University Seoul Hospital (2017-02-002) and Bucheon Hospital (2017-03-004). Informed consent was waived by the Institutional Review Boards as detailed in the Informed Consent Statement below.

### 4.2. miRNA Extraction and Sequencing

Total RNA including small RNAs was extracted from FFPE tissues using the miRNeasy Mini Kit (Qiagen, Hilden, Germany). RNA quantity and quality were assessed by spectrophotometry and an Agilent 2100 Bioanalyzer (Agilent Technologies, Santa Clara, CA, USA). Small RNA sequencing libraries were constructed from 10 ng total RNA per sample using the SMARTer smRNA-Seq Kit (Takara Bio, Shiga, Japan). Library quality was validated on a Bioanalyzer and quantified by qPCR. Pooled libraries were sequenced on an Illumina HiSeq 2500 platform (101 bp single-end reads). Raw data were deposited in NCBI’s Gene Expression Omnibus (Accession: GSE179961).

### 4.3. Bioinformatic Analysis of Differential miRNA Expression

Adapter trimming and quality filtering were performed to retain clean reads ≥ 15 nt. Quality-control procedures included assessment of total sequencing reads, adapter-trimming efficiency, mapping rates to miRBase v21, and library complexity. Samples with more than one million clean reads and mapping rates exceeding 80% were considered suitable for downstream analyses. No sample failed the predefined quality-control criteria. Reads were aligned to the human genome and miRBase v21 to quantify known mature miRNAs. Read counts were normalized using the trimmed mean of M-values (TMM) method and expressed as reads per million (RPM). Differential expression between localized (*n* = 5) and locally advanced (*n* = 4) groups was assessed using the limma/voom pipeline; miRNAs with low counts (undetected in >50% of samples) were excluded. Significance was defined as *p* < 0.05 and fold-change ≥ 2. DESeq2 was used as a complementary approach and yielded concordant results. Hierarchical clustering (pheatmap, complete linkage, Euclidean distance) and volcano plots were generated in R.

### 4.4. Target Gene and Pathway Analysis

Predicted targets of DE-miRNAs were identified using TargetScan (Release 7.2) [24,25] and subsequently cross-referenced with experimentally validated miRNA–target interactions obtained from miRTarBase [26]. High-confidence targets were defined by TargetScan context++ score ≤ −0.4 or strong miRTarBase evidence. KEGG pathway and Disease Ontology enrichment analyses were performed using DAVID bioinformatics resources (v2023) [27] with a significance threshold of *p* < 0.05. Results are presented in Figure 3.

### 4.5. In Silico Validation

Two publicly available mRNA microarray datasets were used for in silico validation: GSE46602 (Affymetrix HG-U133 Plus 2.0) and GSE21034 (Affymetrix Human Exon 1.0 ST), the mRNA subseries of the GSE21032 superseries. The TCGA-PRAD dataset was also analyzed; however, because its available data items did not match the clinical variables used in this study, the GSE datasets were used for the in silico validation. Because GSE46602 and GSE21034 were generated on different microarray platforms, they were not combined; each dataset was quantile-normalized separately using limma and analyzed as an independent validation cohort. Expression levels of CLTC, CDK6, and LASP1 were compared between localized (≤pT2) and locally advanced (≥pT3) tumors within each dataset using the Mann–Whitney U test, and *p*-values were adjusted for multiple testing by the Benjamini–Hochberg procedure. Analyses were performed in R v4.2.1.

### 4.6. Statistical Analysis

Clinical differences between groups were assessed using Student’s *t*-test or the Wilcoxon test for continuous variables and Fisher’s exact test for categorical variables. All tests were two-sided, with *p* < 0.05 considered significant. Analyses were performed in R (v4.0.3) and Prism 8 (GraphPad Software).

## 5. Conclusions

In this exploratory pilot small RNA-seq analysis of nine archival FFPE prostatectomy specimens, we identified seven unique mature miRNAs that differ in expression between localized (pT2) and locally advanced (pT3–T4/N+) prostate cancer. Five unique mature miRNAs (miR-145-5p, miR-205-5p, miR-206, miR-24-1-5p, and hsa-miR-3648) were downregulated in locally advanced disease, and two (hsa-miR-625-3p and miR-324-5p) were upregulated. In silico analysis of public mRNA datasets suggests that CLTC and CDK6, putative downstream targets of the downregulated miRNAs, are overexpressed in locally advanced tumors. These findings are hypothesis-generating. Independent qRT-PCR validation, paired miRNA–mRNA correlation analysis, protein-level confirmation, and functional studies are required before any conclusions regarding biomarker utility or therapeutic relevance can be drawn.

## Figures and Tables

**Table 1:** Clinical and Pathological profiles of Patients.

Variable	Localized PCa (*n* = 5)	Locally Advanced PCa (*n* = 4; pT3–T4/N+)	*p*-Value
Age at surgery (years)	65.0 ± 3.8	73.0 ± 1.1	0.10 (n.s.)
Preoperative PSA (ng/mL)	22.8 ± 15.3	21.2 ± 8.8	0.87 (n.s.)
Pathological stage (categorical)	2.0 ± 0.0 (all pT2)	3.5 ± 0.3 (≥pT3)	<0.01
ISUP Grade Group—see Supplementary Table S1	2.8 ± 0.8 (mostly 3 + 3)	4.3 ± 0.5 (≥Gleason 8)	0.03
Biochemical recurrence	0/5 (0%)	2/4 (50%)	–
Clinical progression	0/5 (0%)	1/4 (25%)	–
Cancer-specific mortality	0/5 (0%)	1/4 (25%)	–

**Note:** Data are presented as mean ± standard error or number of patients. Gleason score is reported as primary + secondary pattern with ISUP Grade Group; see Supplementary Table S1. “Locally Advanced PCa” includes pT3–T4/N+ without distant metastasis. n.s., not significant.

**Table 2:** Key Differentially Expressed miRNAs in Advanced vs. Localized Prostate Cancer. Both nominal *p*-values and Benjamini–Hochberg FDR-adjusted *p*-values are shown.

Mature miRNA	Precursor Locus	log_2_FC (High/Low)	*p*-Value	FDR-Adjusted *p*-Value
hsa-miR-625-3p	hsa-mir-625	3.170	0.018	0.032
hsa-miR-324-5p	hsa-mir-324	2.077	0.024	0.033
hsa-miR-145-5p	hsa-mir-145	−1.061	0.013	0.029
hsa-miR-3648	hsa-mir-3648-1/hsa-mir-3648-2	−2.304	0.047	0.047
hsa-miR-205-5p	hsa-mir-205	−2.529	<0.001	0.003
hsa-miR-206	hsa-mir-206	−2.637	0.005	0.016
hsa-miR-24-1-5p	hsa-mir-24-1	−2.948	0.032	0.038

Note: log_2_FC represents expression change in locally advanced versus localized prostate cancer. Positive values indicate upregulation in locally advanced tumors, whereas negative values indicate downregulation. *p*-values were adjusted for multiple testing using the Benjamini–Hochberg false discovery rate (FDR) procedure. hsa-miR-3648 was detected from two precursor loci but was counted as one unique mature miRNA.

## Data Availability

The original contributions presented in this study are included in the article/Supplementary Materials. Further inquiries can be directed to the corresponding authors.

## Supplementary Materials

The following supporting information can be downloaded at https://www.mdpi.com/article/10.3390/ph19081141/s1, Table S1: Clinicopathological variables included in exploratory analyses; Table S2: Representative miRNA–target gene interactions identified from TargetScan and miRTarBase; Table S3: Representative KEGG pathway enrichment results.

## Abbreviations

The following abbreviations are used in this manuscript:

PSA	Prostate-specific antigen
miRNAs	MicroRNAs
FFPE	Formalin-fixed paraffin-embedded
DE-miRNAs	Differentially expressed microRNAs
PCa	Prostate cancer
EMT	Epithelial–mesenchymal transition
CLTC	Clathrin Heavy Chain
CDK6	Cyclin-Dependent Kinase 6
FDR	False Discovery Rate
TCGA	The Cancer Genome Atlas
GEO	Gene Expression Omnibus
TMM	Trimmed Mean of M-values
KEGG	Kyoto Encyclopedia of Genes and Genomes
