# Supplementary material for: Exploratory Small RNA Sequencing in Localized Versus Locally Advanced Prostate Cancer: Putative Roles of CLTC and CDK6 as Downstream Targets of Differentially Expressed MicroRNAs
Source: Pharmaceuticals (Basel). 2026 Jul 23;19(8):1141. doi: 10.3390/ph19081141 (PMC13516503; doi:10.3390/ph19081141)
Supplement: Supplementary file 1 [file pharmaceuticals-19-01141-s001.zip › pharmaceuticals-4313696-supplementary.pdf]

SUPPLEMENTARY MATERIALS

Supplementary Table S1. Clinicopathological variables included in exploratory analyses.

**Patient ID**	**Group**	**Age**	**PSA**	**Pathologic Stage**	**pN**	**Gleason Score**	**ISUP GG**	**Margin**	**EPE**	**SVI**	**Tumor Cellularity (%)**	**FFPE Year**	**DV200 (%)**	**Follow-up (months)**	**BCR**	**CSM**
L1	Localized	61	8.4	pT2a	N0	3+3	1	Negative	No	No	75	2004	68	84	No	No
L2	Localized	64	11.2	pT2b	N0	3+4	2	Negative	No	No	70	2005	72	76	No	No
L3	Localized	66	15.7	pT2c	N0	3+3	1	Negative	No	No	80	2007	65	69	No	No
L4	Localized	68	39.1	pT2c	N0	4+3	3	Focal Positive	No	No	60	2009	58	52	No	No
L5	Localized	66	39.6	pT2c	N0	3+4	2	Negative	No	No	78	2010	70	61	No	No
A1	Locally Advanced	71	12.5	pT3a	N0	4+4	4	Positive	Yes	No	65	2003	61	48	Yes	No
A2	Locally Advanced	72	19.8	pT3b	N0	4+5	5	Positive	Yes	Yes	70	2006	57	38	No	No
A3	Locally Advanced	74	23.1	pT4	N1	5+4	5	Positive	Yes	Yes	55	2008	52	31	Yes	Yes
A4	Locally Advanced	75	29.4	pT3a	N0	4+3	3	Positive	Yes	No	62	2011	63	42	No	No

Note: PSA, prostate-specific antigen; ISUP GG, International Society of Urological Pathology Grade Group; EPE, extraprostatic extension; SVI, seminal vesicle invasion; BCR, biochemical recurrence; CSM, cancer-specific mortality.

Supplementary Table S2. Representative miRNA–target gene interactions identified from TargetScan and miRTarBase.

miRNA	Target Gene	Evidence Source	Interaction Type
miR-206	CLTC	TargetScan / miRTarBase	Predicted + validated
miR-205-5p	CLTC	TargetScan	Predicted
miR-145-5p	CDK6	TargetScan / miRTarBase	Predicted + validated
miR-24-1-5p	CDK6	TargetScan	Predicted
miR-206	LASP1	miRTarBase	Validated
miR-205-5p	HMGB3	miRTarBase	Validated

Note: Interaction evidence was obtained from TargetScan (Release 7.2) and miRTarBase databases.

Supplementary Table S3. Representative KEGG pathway enrichment results.

**Pathway**	**Gene Count**	**Raw p-value**	**Adjusted p-value (FDR)**	**Representative Target Genes**
PI3K-Akt signaling pathway	18	0.0012	0.009	CDK6, EGFR, VEGFA
MAPK signaling pathway	15	0.0024	0.014	MAPK1, RAF1, FOS
Regulation of actin cytoskeleton	11	0.0041	0.021	CLTC, RAC1, CDC42
Focal adhesion	10	0.0063	0.028	ITGB1, PXN, VEGFA

Note: Adjusted p-values were calculated using the Benjamini–Hochberg false discovery rate correction.
